# No Paradoxical Effect of Smoking Status on Recurrent Cardiovascular Events in Patients Following Percutaneous Coronary Intervention: Thai PCI Registry

**DOI:** 10.3389/fcvm.2022.888593

**Published:** 2022-05-27

**Authors:** Thosaphol Limpijankit, Mann Chandavimol, Suphot Srimahachota, Sukanya Siriyotha, Ammarin Thakkinstian, Rungroj Krittayaphong, Nakarin Sansanayudh

**Affiliations:** ^1^Division of Cardiology, Department of Medicine, Faculty of Medicine, Ramathibodi Hospital, Mahidol University, Bangkok, Thailand; ^2^Division of Cardiovascular Diseases, Department of Medicine, King Chulalongkorn Memorial Hospital, Bangkok, Thailand; ^3^Department of Clinical Epidemiology and Biostatistics, Faculty of Medicine, Ramathibodi Hospital, Mahidol University, Bangkok, Thailand; ^4^Division of Cardiology, Department of Medicine, Siriraj Hospital, Mahidol University, Bangkok, Thailand; ^5^Cardiology Unit, Department of Internal Medicine, Pharmongkutklao Hospital, Bangkok, Thailand

**Keywords:** smoker's paradox, percutaneous coronary intervention (PCI), major adverse cardiovascular events (MACEs), smoking status, cardiovascular prevention

## Abstract

**Background:**

“Smoker's paradox” is a controversial phenomenon that describes an unexpectedly favorable short-term outcome of smokers post-percutaneous coronary intervention (PCI). This study aimed to evaluate the effect of smoking status on recurrent major adverse cardiovascular events (MACEs) in patients who recently underwent PCI and to determine whether it was paradoxical.

**Methods:**

This study utilized data from the nationwide Thai PCI registry, enrolling patients during 2018–2019. Our study factor was smoking status, classified as current smokers, ex-smokers, and nonsmokers. The outcome of interest was the time to occurrence of a composite of MACEs (i.e., all-cause death, myocardial infarction (MI), stroke, and unplanned revascularization) evaluated at about 1-year post-PCI. A propensity score (PS) model using inverse probability weighting with regression adjustment was used to estimate the effect of smoking on the occurrence of MACE.

**Results:**

Current smokers, ex-smokers, and non-smokers accounted for 23, 32, and 45% of the 22,741 subjects, respectively. Smokers were younger, more frequently male, and had fewer traditional atherosclerotic risk factors. Current smokers presented more frequently with ST-elevation MIs (STEMIs) and cardiogenic shock (54 and 14.6%, respectively) than non-smokers. MACE rates were 1.9, 1.2, and 1.6 per 100 patients per month in the current smokers, ex-smokers, and non-smokers, respectively. After applying a PS, patients with a history of current smoking and ex-smoking developed the onset of recurrent MACEs significantly sooner than non-smokers, with a median time of 4.4 vs. 4.9 vs. 13.5 months (*p* < 0.001), respectively.

**Conclusions:**

“Smoker's paradox” was not observed in our patient population. Current smokers and ex-smokers were prone to develop an earlier onset of a post-PCI MACEs than nonsmokers and need a smoke cessation program for further prevention.

## Introduction

Cigarette smoking is the leading preventable cause of premature atherosclerotic coronary artery disease (CAD), which has a strong impact on morbidity and mortality (1, 2). The disastrous effects of tobacco smoke are related to its mixture of more than 7,000 chemicals, which contribute to endothelial dysfunction, inflammation, thrombosis, and oxidation of low-density lipoprotein cholesterol (3). Although this pathophysiology is ultimately reversible, most inflammatory and hemostatic levels may require 5 years to improve after smoking cessation and as long as 20 years to revert to the levels of non-smokers (4).

Many epidemiological studies have found that the long-term prognosis of smokers is far worse than that of non-smokers. However, some studies suggest that there is a “smoker's paradox,” that the outcomes of CAD may be more favorable in smokers than in non-smokers (5–7). This phenomenon was first introduced in the thrombolytic era with reports that smokers with acute myocardial infarction (MI) had lower mortality than non-smokers (6, 8). Similarly, paradoxical associations with smoking were also seen in patients undergoing percutaneous coronary intervention (PCI) for stable CAD and ST-elevation MI (STEMI) (5, 9).

This apparent smoker's paradox may be explained in large part by a cumulative effect of younger age; thus, fewer atherosclerotic risk factors and comorbidities may be present in smokers than in non-smokers at the time of CAD events (5–7, 9, 10).

Researchers have tried to address this possibility by adjusting for confounders and comorbidities at baseline using multivariate logistic regression, but the paradoxical effects of current smokers and ex-smokers often persisted. This might be due to adjustment for confounders only in outcome models (e.g., CAD, death, revascularization, and so on), which may not be sufficient, with residual confounding effects still present.

The ideal study design to balance baseline known and unknown confounders would be a randomized controlled trial (RCT) to prove or disprove the paradoxical effects of smoking, but it would be unethical and impractical to randomly assign participants to smoke or not smoke. Emulation of RCT using a counterfactual propensity score (PS) analysis has been used to assess the possible causal effect of exposure or treatment in observational studies (11). We used this approach to try to prove whether the effect of smoking on the occurrence of major adverse cardiovascular events (MACEs) after PCI is paradoxical or not.

## Materials and Methods

This study used a prospective, multicenter, nationwide Thai PCI registry, which was initiated by the Cardiac Intervention Association of Thailand. The registry protocol, published previously (12), includes data from 39 government and private hospitals that voluntarily participated. All patients enrolled in the study were aged 18 years or older, and received primary or elective PCI during the period from May 2018 to August 2019. The study was approved by the Central Research Ethics Committee of Mahidol University (COA-CREC # 006/2018), along with the Ethics Committee of the Faculty of Medicine, Ramathibodi Hospital (COA-MURA2020/1040). All participants provided written informed consent.

Clinical characteristics, angiographic, and procedural data were retrieved from main electronic registry databases. Subject data included history of cardiovascular risk factors [smoking, hypertension (HT), dyslipidemia, diabetes mellitus (DM), family history of premature CAD], history of underlying diseases and prior treatments [peripheral arterial disease (PAD), cerebrovascular disease (CVD), MI, heart failure, PCI, coronary artery bypass surgery (CABG)], and left ventricular ejection fraction (LVEF). In addition, referral mode (referred or not referred) was considered in the analysis.

Angiographic and procedural data included clinical presentation [STEMI, non-STEMI (NSTEMI)/unstable angina (UA), stable CAD], number of diseased vessels, PCI status (elective, urgent, or emergency), presence of cardiogenic shock, type of contrast agent and volume, access site, coronary lesion characteristics (lesion complexity, in-stent restenosis lesion, bypass graft lesion, ostial lesion, and bifurcation lesion), stent size (length and diameter), plaque modification devices (rotational atherectomy, cutting/scoring balloon, or laser atherectomy), imaging study [intravascular ultrasound study (IVUS) or optical coherence tomography (OCT)], fractional flow reserve wire, and intra-aortic balloon pump (IABP). Perioperative medications, including unfractionated heparin, low-molecular weight heparin (LMWH), glycoprotein IIb/IIIa inhibitor (abciximab and eptifibatide), and P2Y_12_ inhibitors (clopidogrel, prasugrel, and ticagrelor), were also recorded.

“Angiographic success,” was defined as a residual stenosis <20% with stent treatment, or <50% with balloon angioplasty alone. Procedural complications, including death, MI, stroke, cardiogenic shock, heart failure, new requirement of dialysis, blood transfusion, bleeding within 72 h, arrhythmia requiring treatment, endotracheal (ET) intubation, cardioversion/defibrillation, and in-hospital CABG, were also recorded.

### Study Factor

Patients were classified into one of the three groups according to baseline smoking status as follows: non-smokers were patients who had never smoked before the index procedure. Ex-smokers were patients who used to smoke but had quit at least 28 days before the index procedure. Current smokers were patients who continued to smoke until the index procedure.

### Outcomes of Interest

The clinical outcomes of interest were time to development of MACEs after the PCI procedure. MACE was defined as a composite of all causes of death, as well as non-fatal MI, non-fatal stroke, and unplanned revascularization. Death was confirmed by a death certificate from the National Statistics Office, Ministry of Interior. MI was defined as an increase in cardiac troponin (cTn) plus either: (1) evidence of prolonged ischemia as demonstrated by prolonged chest pain (>20 min); or (2) ischemic ST-segment changes or new pathological Q waves; or (3) angiographic evidence of coronary occlusion or no-reflow/slow flow; or (4) imaging evidence of new loss of viable myocardium or new regional wall motion abnormality. Stroke was defined as a new neurological deficit during the first 24 h following PCI secondary to cerebral ischemia or cerebral hemorrhage detected by CT or MRI. Unplanned revascularization was defined as unplanned repeated PCI or CABG. MI during the first 48 h following revascularization was defined as an increase in cTn to >5 × 99th percentile of the upper reference limit (in patients with normal baseline cTn concentrations) or an increase of 20% (in patients with elevated cTn before PCI or CABG).

### Statistical Analysis

Baseline characteristics were described among smoking status groups using mean ± standard deviation (SD) for continuous variables and percentage for categorical variables. Those baseline characteristics were then compared among the three smoking groups using an one-way analysis of variance (ANOVA), quartile regression, or Chi-squared test, as appropriate.

Time to MACE development was calculated by subtracting the date on the last follow-up from the date of the PCI procedure. Patients were censored at the date of last visit if they were lost to follow-up or free of MACE. The effect of smoking on time to MACE development was assessed using an inverse probability weighting and regression adjustment (IPWRA) as follows: first, a multi-logit equation was applied to estimate a PS by regressing smoking (i.e., current smokers and ex-smokers vs. non-smokers) on variables, which might be associated with smoking and also the MACE outcome, as recommended by Austin et al. (13, 14), including demographic data [i.e., age, sex, and body mass index (BMI)], comorbidities (i.e., HT, diabetes, dyslipidemia, PAD, prior-CABG, known CAD, CKD, and CVD), CAD presentations (i.e., STEMI, NSTEMI/UA, and stable CAD), PCI status (i.e., elective, urgent, and emergency), cardiogenic shock at start of PCI, receipt of clopidogrel, disease vessel (left main vs. non-left main), number of lesions, and PCI center characteristics [i.e., number of PCI procedures/cardiologist/year, number of PCI procedures/hospital/year, and experience (years) of cardiologists]. Significant variables were finally kept in the PS model and balanced among the three smoking groups if their weighted standardized mean differences did not exceed 0.2 and variance ratio was close to 1 (15). The density distributions of those variables were also plotted to make sure that the probabilities of being current smokers, ex-smokers, or non-smokers overlapped.

Second, a Weibull survival regression was used to construct the outcome and censor models weighted by the inverse PS of smoking status. Some confounders were also considered, but only significant confounders were finally kept in these two survival models [demographic data (i.e., age and sex), referred case, heart rate on admission, cardiogenic shock at the start of PCI, PCI status (i.e., elective, urgent, and emergency), dialysis for end-stage renal disease (ESRD), CAD presentations (i.e., STEMI, NSTEMI/UA, and stable CAD), arrhythmia requiring treatment, crossover arterial access, disease vessel (left main vs. non-left main), new requirement of dialysis, tamponade, IABP mechanical ventilator support, ET tube intubation, procedural complication, and the result of PCI (failure vs. success)].

Finally, the potential outcome mean (POM, i.e., median time of MACE development) was estimated for each smoking status group. An average treatment effect (ATE), or difference of median time along with 95% confidence interval (CI), was estimated. Furthermore, a hazard ratio (HR) along with 95% CI of smoking status with adjusted PS was estimated. All analyses were performed with STATA version 17.0 (Stata Corp., College Station, TX, USA). A value of *p* < 0.05 was considered statistically significant.

## Results

### Baseline Characteristics of Overall Data

Of the 22,741 study subjects, current smokers, ex-smokers, and non-smokers accounted for 23, 32, and 45% of the total, respectively. Current smokers averaged ~ 5 and ~7 years younger than ex-smokers and non-smokers (mean ages of 59.3, 64.6, and 66.3 years, respectively; *p* < 0.001). Those smokers were more frequently male, with lower BMI and fewer traditional atherosclerotic risk factors (i.e., DM, HT, and dyslipidemia) than non-smokers and ex-smokers. Smokers were also less likely to have other comorbid diseases (i.e., CKD, CVD, and PAD), prior heart failure, and revascularization (i.e., PCI or CABG) (see Table 1).

**Table 1:** Baseline clinical and angiographic characteristics of 22,741 patients undergoing PCI, grouped by smoking status.

**Characteristics**	**Smoking status**			***P*-value**
	**Current smoker**	**Ex-smoker**	**Never**	
	***n* = 5,285**	***n* = 7,239**	***n* = 10,217**	
Age, (years), mean (SD)	59.3 (11.5)	64.6 (11.1)	66.3 (11.5)	<0.001
Male sex, *n* (%)	4,959 (93.8)	6,759 (93.4)	3,983 (39.0)	<0.001
BMI, kg/m^2^, mean (SD)	23.9 (4.1)	24.3 (4.1)	24.5 (4.3)	<0.001
DM, *n* (%)	1,877 (35.5)	2,971 (41.0)	5,202 (50.9)	<0.001
CKD, *n* (%)	1,136 (21.5)	2,316 (32.0)	3,948 (38.6)	<0.001
HT, *n* (%)	2,424 (45.9)	5,152 (71.2)	7,747 (75.8)	<0.001
Dyslipidemia, *n* (%)	2,592 (49.0)	5,062 (69.9)	7,209 (70.6)	<0.001
Cerebrovascular disease, *n* (%)	202 (3.8)	471 (6.5)	623 (6.1)	<0.001
Family history of premature CAD, *n* (%)	356 (6.7)	756 (10.4)	946 (9.3)	<0.001
PAD, *n* (%)	67 (1.3)	131 (1.8)	191 (1.9)	0.017
Prior MI, *n* (%)	817 (15.5)	2,192 (30.3)	2,358 (23.1)	<0.001
LVEF, mean (SD)	49.4 (14.8)	51.3 (15.6)	52.7 (15.7)	0.001
Chronic lung disease, *n* (%)	211 (4.0)	323 (4.5)	207 (2.0)	<0.001
Prior heart failure, *n* (%)	412 (7.8)	1,108 (15.3)	1,611 (15.8)	<0.001
Prior PCI, *n* (%)	736 (13.9)	2,859 (39.5)	3,142 (30.8)	<0.001
Previous CABG, *n* (%)	14 (0.3)	142 (2.0)	207 (2.0)	<0.001
**CAD presentation**, ***n*** **(%)**				
STEMI	2,855 (54.0)	1,265 (17.5)	2,253 (22.1)	<0.001
NSTEMI/unstable angina	1,392 (26.3)	2,080 (28.7)	3,334 (32.6)	
Stable CAD	1,038 (19.6)	3,894 (53.8)	4,630 (45.3)	
Thrombolytic treatment^*^, *n* (%)	1,030 (36.2)	466 (37.3)	727 (32.5)	0.005
Time before thrombolytics, minute, median (IQR)	57 (32, 95)	54 (30, 90)	60 (37, 105)	0.151
Clopidogrel, *n* (%)	4,532 (87.7)	6,380 (90.1)	8,721 (87.8)	<0.001
Unfractionated heparin, *n* (%)	4,673 (88.7)	6,708 (92.8)	9,345 (91.6)	<0.001
GPIIb/IIIa inhibitor (%)	571 (10.8)	304 (4.2)	562 (5.5)	<0.001
**PCI Status**, ***n*** **(%)**				
Emergency	2,309 (43.7)	991 (13.7)	1,988 (19.5)	<0.001
Urgent	1,099 (20.8)	904 (12.5)	1,524 (14.9)	
Elective	1,877 (35.5)	5,344 (73.8)	6,705 (65.6)	
Referred case, *n* (%)	3,661 (69.3)	3,674 (50.8)	5,015 (49.1)	<0.001
Door to balloon, hours, median (IQR)	4.4 (2.4, 11.0)	4.9 (2.4, 16.0)	4.6 (2.4, 10.7)	0.055
Cardiogenic shock at start of PCI, *n* (%)	771 (14.6)	332 (4.6)	709 (6.9)	<0.001
Total volume of contrast (ml), mean (SD)	103.9 (48.1)	114.2 (56.9)	109.3 (53.5)	<0.001
IABP, *n* (%)	279 (5.3)	164 (2.3)	329 (3.2)	<0.001
Radial access, *n* (%)	2,746 (52.0)	3,109 (42.9)	4,207 (41.2)	<0.001
**Extent of CAD**, ***n*** **(%)**				
SVD	1,863 (35.2)	1,562 (21.6)	2,586 (25.3)	<0.001
DVD	1,602 (30.3)	2,114 (29.2)	2,814 (27.5)	
TVD	1,349 (25.5)	2,640 (36.5)	3,505 (34.3)	
Left main	471 (8.9)	923 (12.8)	1,312 (12.8)	
**Lesion characteristic**	*n* = 6,234	*n* = 9,182	*n* = 12,830	
**Lesion complexity**, ***n*** **(%)**				
A	315 (5.1)	492 (5.4)	697 (5.5)	0.462
B1	1,091 (17.6)	1,582 (17.3)	2,305 (18.1)	
B2 or C	4,784 (77.3)	7,047 (77.3)	9,732 (76.4)	
Ostium lesion, *n* (%)	534 (8.6)	1,128 (12.3)	1,532 (12.0)	<0.001
Bifurcation lesion, *n* (%)	696 (11.2)	1,365 (15.0)	1,794 (14.1)	<0.001
Plaque modification, *n* (%)	108 (1.7)	474 (5.2)	744 (5.8)	<0.001
Average stent length per lesion (mm), mean (SD)	24.7 (7.5)	24.3 (7.8)	23.9 (7.9)	<0.001
Average stent diameter per lesion (mm), mean (SD)	3.1 (0.5)	3.0 (0.5)	2.9 (0.5)	0.366

*BMI, body mass index; CABG, coronary artery bypass surgery; CAD, coronary artery disease; CKD, chronic kidney disease; DM, diabetes mellitus; DVD, double vessel disease; HT, hypertension; IABP, intraaortic balloon pump; LVEF, left ventricular ejection fraction; MACE, major adverse cardiac event; MI: myocardial infarction; NSTEMI, non-ST-elevation myocardial infarction; PAD, peripheral arterial disease; PCI, percutaneous coronary intervention; SD, standard deviation; STEMI, ST-elevation myocardial infarction; SVD, single vessel disease; TVD, triple vesseldisease*.

* *For STEMIonly*.

Current smokers presented more frequently with STEMI (54%), cardiogenic shock (14.6%), and required IABP support (5.3%) than the other two groups. In current smokers who presented with STEMI, a thrombolytic agent was given in 36.2%, with the majority using streptokinase (98.5%). Median times before thrombolytic and door to balloon were 57 min (32, 95) and 4.4 h (2.4, 11.0), respectively. Current smoking subjects were more likely to have singular value decomposition (SVD) involvement, less frequently had ostial or bifurcation involvement, and required less plaque modification treatment (Table 1). The most commonly used (more than 85%) antithrombotic regimen in all patients was a combination of clopidogrel and unfractionated heparin. GP IIb/IIIa inhibitors were given more commonly to current smokers than to ex-smokers and non-smokers (10.8 vs. 4.2 vs. 5.5%, respectively; *p* < 0.001).

### Major Adverse Cardiovascular Events

About 97% of the patients in our study were followed up for 18 months with a median of 12 ± 3.7 months. The post-PCI MACE rate was the highest in current smokers, followed by non-smokers and ex-smokers, with incidence rates of 1.9, 1.6, and 1.2 per 100 persons per month, respectively (*p* < 0.001) (Table 2). After adjusting for covariates from the baseline PCI procedure using Weibull survival regression, HRs (95% CI) of current smokers and ex-smokers vs. non-smokers were 0.92 (0.85, 0.99) and 0.90 (0.83, 0.98), respectively; subjects who were current smokers and ex-smokers had an approximately 8 and 10% lower risk of developing MACE than non-smokers (see Supplementary Table S1).

**Table 2:** Estimation of MACE development to smoking and other risk factors: A univariate analysis.

**Characteristic**	**MACE Yes**	**Time at risk (months)**	**Incidence /month**	**HR (95%CI)**	***P*-value**
	***n* = 3,803**				
**Smoking status**, ***n*** **(%)**					
Current smoker	1,051	56942.5	0.0185	1.2 (1.1, 1.3)	<0.001
Ex-smoker	974	84643.9	0.0115	0.6 (0.7, 0.8)	<0.001
Never	1,778	114590.3	0.0155	1	
Age, years, mean (SD)	67.4 (12.6)	-	-	1.026 (1.023, 1.029)	<0.001
**Gender**, ***n*** **(%)**					
Female	1,356	77566.5	0.0175	1.3 (1.2, 1.3)	<0.001
Male	2,447	178610.3	0.0137	1	
**Referred case**, ***n*** **(%)**					
Yes	2,376	135354.8	0.0176	1.4 (1.2, 1.5)	<0.001
No	1,427	120822.0	0.0118	1	
BMI, kg/m^2^, mean (SD)	23.5 (4.3)	-	-	0.947 (0.939, 0.954)	<0.001
**CKD**, ***n*** **(%)**					
Yes	1,947	76044.8	0.0256	2.3 (2.2, 2.5)	<0.001
No	1,856	180131.9	0.0103	1	
**HT**, ***n*** **(%)**					
Yes	2,562	173380.0	0.0148	0.99 (0.92, 1.06)	0.733
No	1,241	82796.8	0.0150	1	
**Dyslipidemia**, ***n*** **(%)**					
Yes	2,099	172405.1	0.0122	0.6 (0.5, 0.7)	<0.001
No	1,704	83771.7	0.0203	1	
**Prior MI**, ***n*** **(%)**					
Yes	737	61954.4	0.0119	0.8 (0.7, 0.9)	<0.001
No	3,066	194222.4	0.0158	1	
**Prior heart failure**, ***n*** **(%)**					
Yes	839	31967.4	0.0262	1.9 (1.7, 2.0)	<0.001
No	2,964	224209.4	0.0132	1	
**Prior PCI**, ***n*** **(%)**					
Yes	790	79800.9	0.0098	0.60 (0.55, 0.65)	<0.001
No	3,013	176375.8	0.0171	1	
**Cerebrovascular disease**, ***n*** **(%)**					
Yes	1,995	13337.5	0.0251	1.7 (1.5, 1.9)	<0.001
No	1,808	242839.2	0.0143	1	
**DM**, ***n*** **(%)**					
Yes	119	109959.1	0.0181	1.4 (1.3, 1.5)	<0.001
No	3,684	146217.6	0.0124	1	
**PAD**, ***n*** **(%)**					
Yes	839	3865.7	0.0308	2.0 (1.6, 2.4)	<0.001
No	2,964	252311.0	0.0146	1	
**CAD presentation**, ***n*** **(%)**					
STEMI	1,747	62789.2	0.0278	3.4 (3.1, 3.6)	<0.001
NSTEMI/unstable angina	1,182	77173.9	0.0153	2.0 (1.8, 2.2)	<0.001
Stable CAD	874	116213.5	0.0075	1	
**Extent of CAD**, ***n*** **(%)**					
Left main	630	28797.7	0.0219	1.4 (1.3, 1.6)	<0.001
TVD	1,161	85733.7	0.0135	0.9 (0.8, 1.0)	0.072
DVD	1,020	74026.9	0.0138	0.9 (0.9, 1.0)	0.163
SVD	992	67618.4	0.0147	1	
**PCI Status**, ***n*** **(%)**					
Emergency	1,727	48,866.8	0.0353	3.7 (3.5, 4.0)	<0.001
Urgent	670	38,598.4	0.0174	2.0 (1.8, 2.2)	<0.001
Elective	1,406	168711.6	0.0083	1	
**Cardiogenic shock at start of PCI**, ***n*** **(%)**					
Yes	896	13,030.0	0.0688	4.5 (4.2, 4.8)	<0.001
No	2,907	243,146.8	0.0120	1	
**IABP**, ***n*** **(%)**					
Yes	463	4,497.5	0.1029	5.3 (4.8, 5.9)	<0.001
No	3,313	250,064.0	0.0132	1	
**Radial access**, ***n*** **(%)**					
Yes	1,519	114,624.4	0.0133	0.8 (0.7, 0.9)	<0.001
No	2,284	141,552.4	0.0161	1	
**Lesion complexity**, ***n*** **(%)**					
B2 or C	3,186	204,530.3	0.0156	1.5 (1.2, 1.8)	<0.001
B1	471	38,898.0	0.0121	1.2 (1.0, 1.4)	0.115
A	117	11,414.7	0.0102	1	

*Abbreviations as in Supplementary Table 1*.

An IPWRA was applied to balance the covariates among the three smoking status groups (see Table 3). Standardized absolute mean differences before weighting by PS ranged from 0.0601 to 0.6455 for current smokers vs. non-smokers, and 0.0021 to 1.4038 for ex-smokers vs. nonsmokers. After weighting by PS, the weighted standardized mean differences of those two corresponding comparisons ranged from 0.006 to 0.026 and 0.00001 to 0.0178, which were far <0.2. In addition, the variance ratios of covariates were close to 1, i.e., from 0.9857 to 1.1654 and 0.9002 to 1.1157, respectively. This indicated that all covariates were well balanced between current smokers vs. non-smokers and ex-smokers vs. non-smokers. In addition, density plots indicated that the distributions of each covariate after weighted balancing were very close (see Supplementary Figure S1).

**Table 3:** Balance of factors associated with smoking status.

**Estimation**	**Standardized differences**		**Variance ratio**	
	**Before weighted**	**After weighted**	**Before weighted**	**After weighted**
**Current smoker vs never smoke**
Age	−0.6081	0.0215	1.0120	0.9875
Female vs Male	−1.4273	0.0081	0.2424	1.0067
BMI	−0.1456	−0.0100	0.9129	1.1654
Hypertension vs. none	−0.6455	0.0034	1.3551	0.9975
Dyslipidemia vs. none	−0.4496	−0.0243	1.2035	1.0149
CKD vs. none	−0.3789	0.0188	0.7132	1.0137
**Disease vessel**				
DVD vs. SVD	0.0601	0.0075	1.0577	1.0069
TVD vs. SVD	−0.1923	0.0260	0.8438	1.0182
Left main vs. SVD	−0.1260	−0.0060	0.7262	0.9857
**Ex-smoker vs never smoker**
Age	−0.1508	−0.0040	0.9263	0.9002
Female vs. Male	−1.4038	0.0020	0.2609	1.0016
BMI	−0.0469	0.0030	0.9003	1.1157
Hypertension vs. none	−0.1056	0.0178	1.1194	0.9867
Dyslipidemia vs. none	−0.0128	0.00001	1.0114	1.0000
CKD vs. none	−0.1393	−0.0056	0.9177	0.9958
**Disease vessel**				
DVD vs. SVD	0.0369	0.0045	1.0361	1.0041
TVD vs. SVD	0.0455	0.0130	1.0283	1.0093
Left main vs. SVD	−0.0021	0.0009	0.9954	1.0022

*Abbreviations as in Supplementary Table 1*.

Median times to the development of MACE were estimated to have median times of 4.4 (2.9, 5.9), 4.9 (3.4, 6.4), and 13.5 (8.6, 18.4) months in current smoker, ex-smoker, and non-smoker groups, respectively (see Table 4). The differences in medians were −9.1 (−14.2, −4.0) and −8.7 (−13.6, −3.7), indicating that the time to post-PCI MACE occurrence was 9.1 and 8.7 months sooner in patients who continued to smoke and in ex-smokers relative to those who had never smoked. In other words, the times to MACEs were about 0.3 and 0.4 times shorter in current smokers and ex-smokers than in non-smokers, respectively.

**Table 4:** Time to MACE among smoking groups: a propensity score model.

**Treatment**	**Median time (month)**	**Lower limit**	**Upper limit**	**ATE (95%CI)**
Current smoker	4.4	2.9	5.9	−9.12 (−14.23, −4.02)
Ex-smoker	4.9	3.4	6.4	−8.65 (−13.58, −3.73)
Never-smoker	13.5	8.6	18.4	0

A sub-group analysis by gender was also performed, the median time to MACE developments in men who were current smokers, ex-smokers, and non-smokers were 3.5 (2.5, 4.4), 5.6 (3.9, 7.4), and 13.9 (12.6, 15.4), respectively. This could be interpreted that male current smokers and ex-smokers were about −10.5 (−12.2, −8.8) and −8.3 (−10.4, −6.3) months sooner to develop MACEs than non-smokers. The time to develop MACE was shorter in women than in men, with a median time to the corresponding smoking statuses of 2.4 (0.9, 3.9), 2.7 (1.4, 4.1), and 6.3 (4.5, 8.1) months, respectively; i.e., current and ex-smoking women are about −3.9 (−6.2, −1.5) and −3.6 (−5.8, −1.3) months sooner, respectively, to develop MACEs than non-smokers.

The positivity assumption was checked with overlap plots (see Supplementary Figure S2), which indicated that the probabilities of the three smoking status groups very much overlapped, and that patients in each group still had positive probabilities of being in each group. In addition, the censor probability plots indicated that the groups were very close to each other (see Supplementary Figure S3).

## Discussion

To determine if the effect of smoking on the occurrence of MACE was paradoxical, we performed a PS analysis using a large-scale nationwide cohort from the Thai PCI registry. Our findings indicated that about 50% of current smokers and ex-smokers developed earlier MACEs about 4 and 5 months after PCI, whereas this was about 13 months for nonsmokers. Thus, both current smokers and ex-smokers tended to develop MACEs about 9 months sooner than non-smokers.

“Smoker's paradox” is still a controversial issue. The data analytic methods used in previous studies may have played a role in concluding that there was a paradoxical effect. The best approach to prove the effects of smoking would be an RCT study design, in which known and unknown confounders can be well-balanced and controlled (11), but such a design cannot be used for smoking status for ethical reasons. Previous studies (5–10) applied multivariate analyses using logistic or Cox regression analyses in which all relevant confounders were adjusted in only one equation (i.e., the outcome model). This did not guarantee that the confounders were balanced among smoking status groups. As a result, a “paradoxical” effect of smoking was still present. Similar to our study, after conventional adjustment for confounders in the Weibull survival regression model, the paradoxical effects of current smokers and ex-smokers were still present with 8 and 10% lower risk of MACEs, respectively. In contrast, we used a PS analysis to consider not only the outcome, but also treatment (in this case, smoking status) models to properly balance confounders that are associated with smoking status or outcome between smoking status groups. As a final result, the “paradoxical” effect of smoking disappeared in our findings.

An ethnic difference might be present in the “smoker's paradox” (16, 17). This may be explained by major epidemiologic differences between Western and Asian populations, such as the amount of smoking, age of smoking initiation, the use of filtered or mild tobacco, and genetics of cytochrome metabolism, so we cannot conclude to what extent those factors might explain this phenomenon as the relevant genetic differences have not been sufficiently elucidated.

Our registry makes clear that cigarette smoking continues to be a major health hazard in Thailand and contributes significantly to cardiovascular mortality and morbidity. There was a substantial proportion of Thai patients who were undergoing PCI and smoked cigarettes. Almost one-quarter of patients were smokers at the time of the index procedure and more than half of patients had smoked previously. About 80% of the current smokers in our study presented with an acute coronary syndrome, predominately STEMI (54%). This finding is consistent with previous pathologic studies reporting that cigarette smoking increases the risk of plaque rupture and acute thrombosis (18, 19). Importantly, there might have been some current smokers who developed sudden cardiac arrest after MI before medical contact, as previously reported (18, 20) although there were no available data for patients who died due to CAD prior to the PCI procedure. In this situation, primary prevention is the best way to avoid this disastrous event.

As in previous studies, current smokers in our study were younger, had fewer risk factors and comorbidities, and less extensive CAD than exsmokers and non-smokers (10, 21, 22). They also more frequently experienced heart failure, cardiogenic shock, and in-hospital death and MACEs. These adverse consequences differ from previous studies that demonstrated a smoker's paradox in patients with STEMI who had significantly lower risk-adjusted in-hospital mortality after treatment with fibrinolytics or PCI (5, 23). There are several possible explanations for the absence of “paradoxical” effects, such as the differences in the use of thrombolytics, primary PCI, and delayed medical contact with the balloon during transfer from the primary center to the catheterization laboratory. Patients who are current smokers usually have a greater thrombus burden, leading to greater efficacy of thrombolytic and antiplatelet therapies. Multiple randomized trials with thrombolytic therapies report lower short- and long-term mortality in smokers with STEMI (24–26). However, the benefit of fibrinolytics is achieved when they are administered early and restore coronary artery patency. The median duration from the first medical contact to thrombolytic treatment in our registry subjects was about 50–60 min, which was still longer than guideline recommendations (27, 28). This data suggests that a pharmacoinvasive strategy is suitable for the treatment of current smokers who develop STEMI in rural areas and cannot be transferred to primary PCI centers within 120 min, as recommended in the guidelines. This aggressive beneficial approach was supported by the recent publication of the smoking paradox in patients with ischemic stroke who were treated with intra-arterial thrombolysis in combination with mechanical thrombectomy (29).

The analysis of gender-related differences in the risk of smoking-related PCI in our study demonstrated that, although smoking was more prevalent in men than women in our data (i.e., 69 vs. 31%), its effects on the development of MACEs were shorter in women than in men. For instance, women who were current smokers would take about 2.4 months whereas men would take about 3.5 months. The impact of smoking as a risk factor for CAD may be more detrimental in postmenopausal women because of the lack of natural estrogen protection, an unfavorable lipid profile, or in subjects at risk of thrombosis such as women taking oral contraceptives (30, 31). This finding is relevant to make the population aware of the impact of smoking on health.

Many interactions between tobacco smoke and medications have been identified (32). In our cohort, the commonly used antiplatelet agent was clopidogrel. Tobacco increases the metabolism of clopidogrel to its active metabolite by the induction of CYP1A2. The effect of clopidogrel is enhanced in smokers (>10 cigarettes/day), with significant platelet inhibition, and decreased platelet aggregation. From the analysis of large trials on PCI in acute coronary syndrome with clopidogrel (33–35), the long-term prognosis of smokers treated with clopidogrel was better than that of non-smokers. However, this benefit did not appear in our patients, as current smokers and ex-smokers still developed recurrent MACEs earlier than non-smokers. This finding could be explained by the fact that inflammatory markers and hypercoagulable state may persist for a longer period even after smoking cessation. Non-compliance with dual antiplatelet therapy and persistence of cigarette smoking are also the possibilities to explain this earlier onset of recurrent events. In addition, smoking cessation in patients treated with clopidogrel after PCI might be associated with increased platelet reactivity and a greater risk of high platelet reactivity, the so-called “smoking cessation paradox” (36, 37). This phenomenon may increase the risk of thrombotic complications in patients treated with clopidogrel. There is evidence that current cigarette smoking is an independent risk factor for subacute stent thrombosis (38). Doubling the dose of clopidogrel (39) or switching to more potent P2Y_12_ inhibitors (40, 41) may overcome a potential smoking cessation paradox in patients who stopped smoking after PCI. Further studies are required to determine the optimal antiplatelet strategy for stented patients who effectively quit smoking during clopidogrel treatment. Lastly, smoking cessation may increase body weight and cause a dysmetabolic profile (42) although these changes cannot explain the earlier onset of MACEs, because it is too soon. Our findings for ex-smokers also did not show a paradoxical effect relative to non-smokers.

Based on the recent evidence, the concept of smoker's paradox becomes inconclusive. After adjusting for baseline characteristics, this pseudo-paradox does not exist. This pathogenesis is a multifactorial disorder involving inflammation, plaque rupture, augmented thrombotic factors, hepatic enzyme induction, and drug interaction and compliance. Cigarette cessation should be encouraged in all patients before and after PCI.

### Limitations of This Study

Although our study was a well-designed prospective cohort study, some limitations could not be avoided. First, a history of cigarette smoking before PCI was used for analysis; it was not known whether these subjects continued smoking or quit smoking. In addition, descriptive smoking data, including the duration of smoking, number of cigarettes per day or pack-year, and the time from stop smoking to the index procedure, were not available. Moreover, the separation of smokers and ex-smokers could be arbitrary as the biological effects of smoking are long lasting. Similarly, the separation between non-smokers and current smokers could not be completely carried out because of atmospheric pollution and passive smoking. Second, compliance with relevant treatments (i.e., antiplatelet, statin, beta-blocker, angiotensin receptor blockers, etc.), dietary pattern (including herb and alcohol), and other risk factors of MACEs may have changed over time after PCI procedures, but only the baseline data were considered in the analyses. Currently, the dietary pattern itself could contribute to prothrombotic and inflammatory effects, and that background could confound our results. Lastly, the causes of death were documented by the death certificate and by the cardiologists of the participating sites without being re-adjudicated by an expert committee. For this reason, we considered all causes of death instead of CVD deaths. However, the majority of MACEs in current smokers were driven by MI and stroke (fatal and non-fatal) and unplanned revascularization (64.7%).

## Conclusion

In summary, there was no actual “smoker's paradox” found in our post-PCI population when we applied a PS analysis. Current smokers and ex-smokers were prone to develop an earlier onset of post-PCI MACEs than non-smokers. The cessation of smoking should be re-emphasized to reduce the risk of recurrent MACE development.

## Data Availability Statement

The raw data supporting the conclusions of this article will be made available by the authors, without undue reservation.

## Ethics Statement

The studies involving human participants were reviewed and approved by Ethics Committee of the Faculty of Medicine, Ramathibodi Hospital (COA-MURA2020/1040). The patients/participants provided their written informed consent to participate in this study.

## Author Contributions

TL: research proposal, applying for ethic committee, and writing manuscript. MC: investigator, data management, and comments of manuscript. SSr: investigator and data management. SSi: statistical analysis. AT: supervision of statistical analysis and editing manuscript. RK: providing comments and suggestion of manuscript. NS: primary investigator of project. All authors contributed to the article and approved the submitted version.

## Funding

This project received a research grant from the Health System Research Institute, the Ministry of Public Health, Bangkok, Thailand.

## Conflict of Interest

The authors declare that the research was conducted in the absence of any commercial or financial relationships that could be construed as a potential conflict of interest.

## Publisher's Note

All claims expressed in this article are solely those of the authors and do not necessarily represent those of their affiliated organizations, or those of the publisher, the editors and the reviewers. Any product that may be evaluated in this article, or claim that may be made by its manufacturer, is not guaranteed or endorsed by the publisher.

## Supplementary Material

Supplementary Figure 1 Balance plot. This produces kernel density plots of each covariate by smoking status groups.

Supplementary Figure 2 Overlapping plot. This plots the estimated densities of the probability of being each smoking status to see if each individual patient has a positive probability of being each smoking status.

Supplementary Figure 3 Probability of censor by smoking groups. Plot probability of censor on y-axis and time on x-axis. The three curves line closed together indicating the three smoking status groups have a similar probability of being censored.

Supplementary Table 1 Factors associated with MACE: A multivariate Weibull survival regression.
