# Supplementary figures and images for: No Paradoxical Effect of Smoking Status on Recurrent Cardiovascular Events in Patients Following Percutaneous Coronary Intervention: Thai PCI Registry
Source: Front Cardiovasc Med. 2022 May 27;9:888593. doi: 10.3389/fcvm.2022.888593 (PMC9197099; doi:10.3389/fcvm.2022.888593)

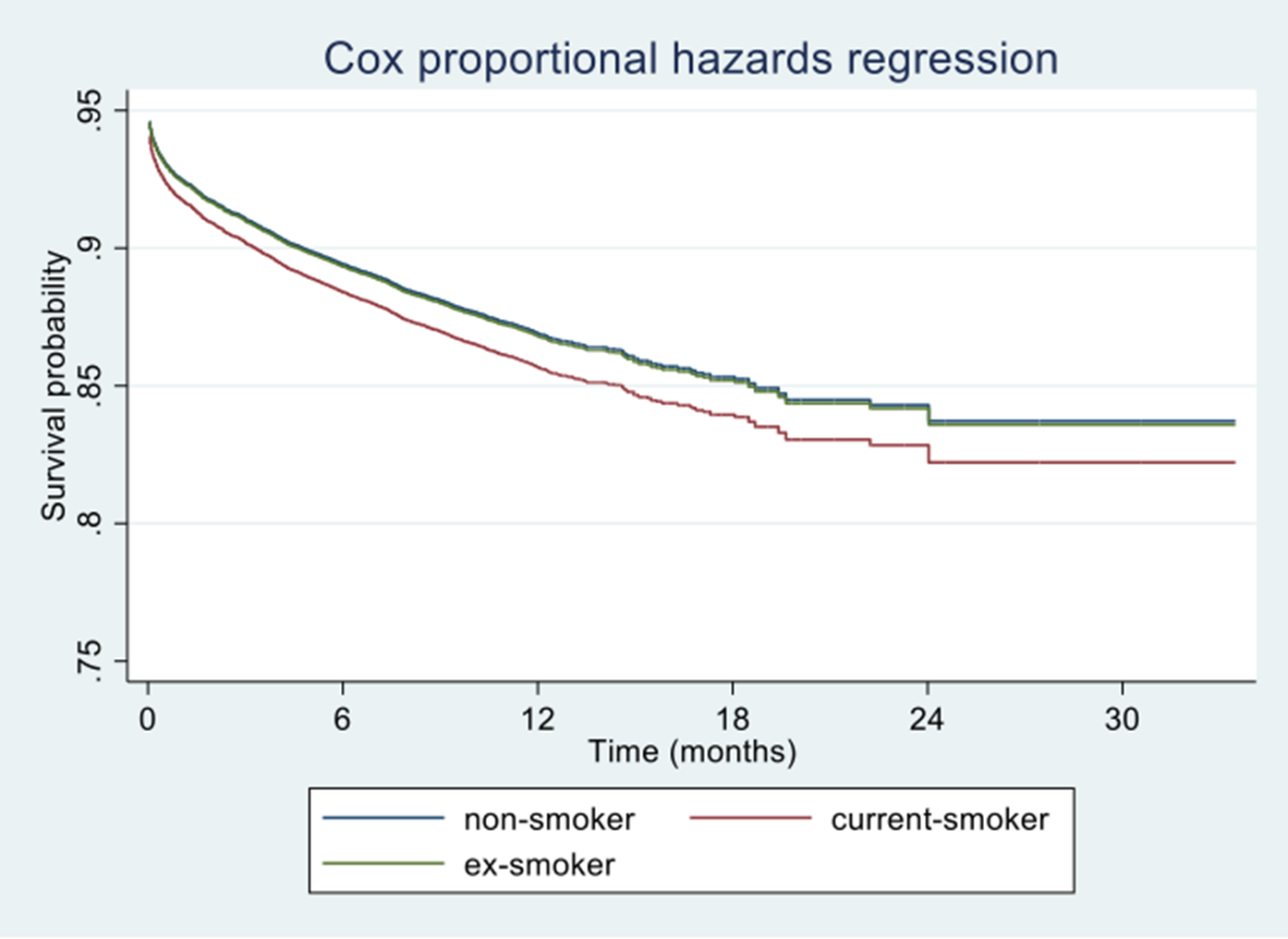

Supplement: Supplementary Figure 1 — Balance plot. This produces kernel density plots of each covariate by smoking status groups. [file Image_1.PNG]

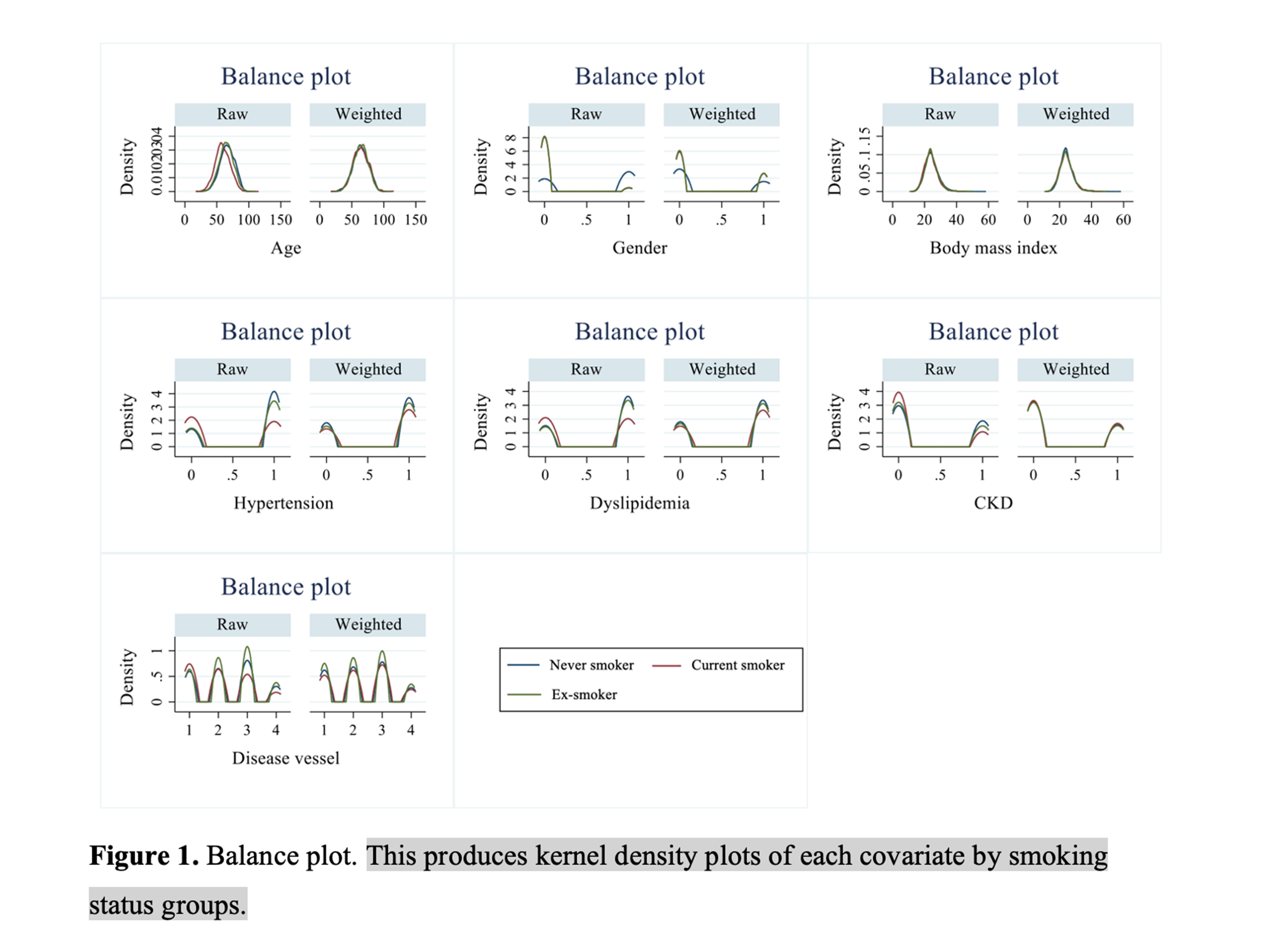

Supplement: Supplementary Figure 2 — Overlapping plot. This plots the estimated densities of the probability of being each smoking status to see if each individual patient has a positive probability of being each smoking status. [file Image_2.PNG]

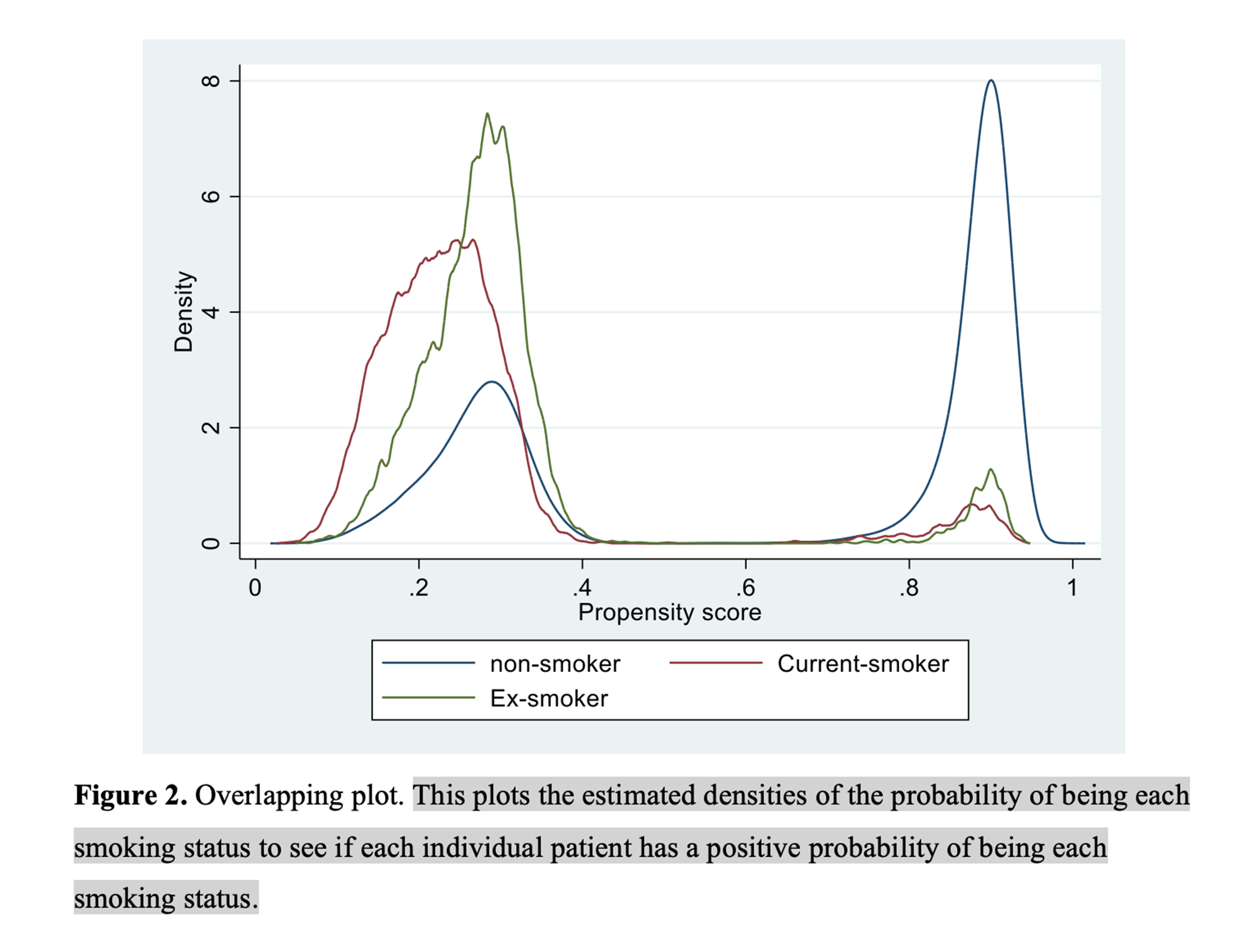

Supplement: Supplementary Figure 3 — Probability of censor by smoking groups. Plot probability of censor on y-axis and time on x-axis. The three curves line closed together indicating the three smoking status groups have a similar probability of being censored. [file Image_3.PNG]

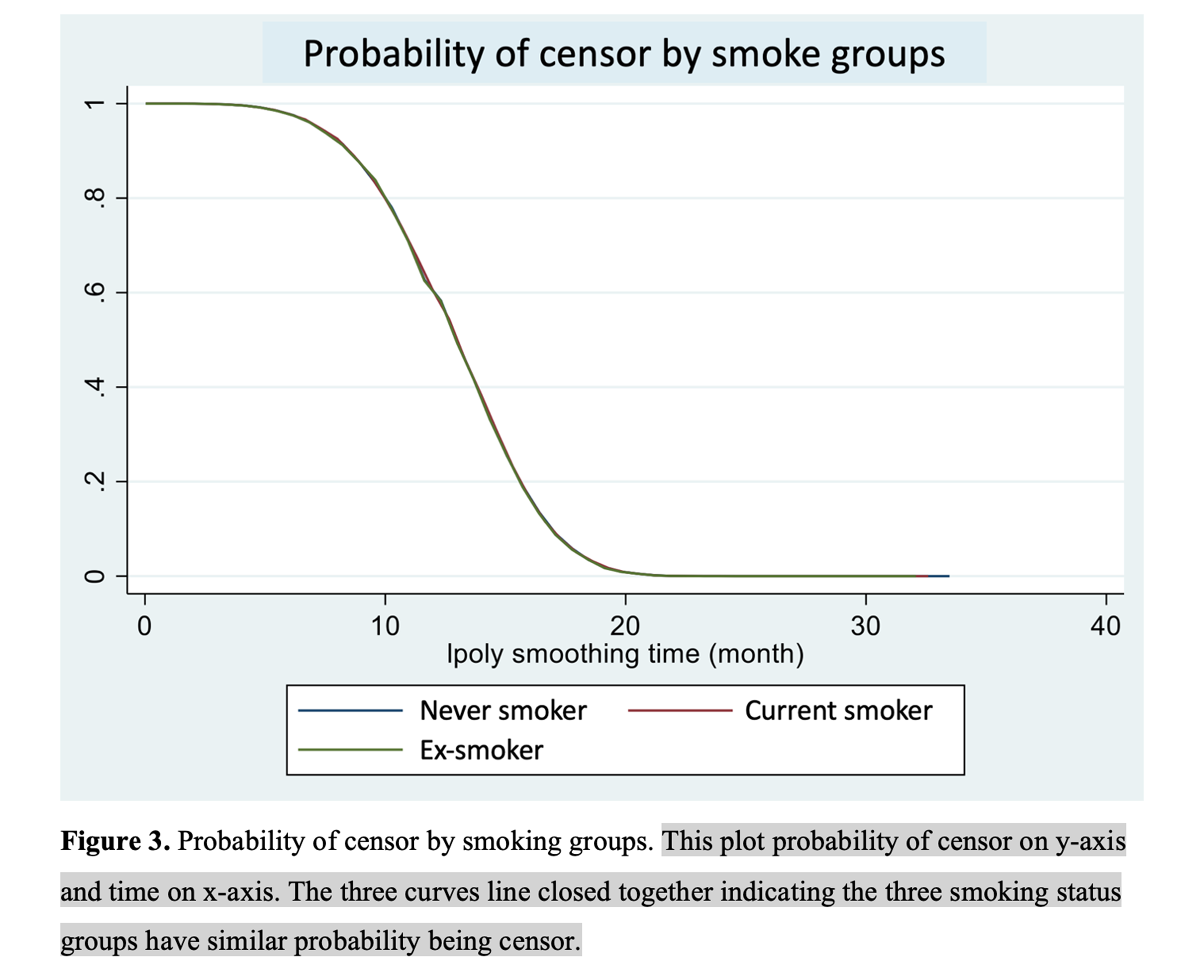

Supplement: Supplementary file 5 [file Image_4.PNG]
