# Supplementary material for: No Paradoxical Effect of Smoking Status on Recurrent Cardiovascular Events in Patients Following Percutaneous Coronary Intervention: Thai PCI Registry
Source: Front Cardiovasc Med. 2022 May 27;9:888593. doi: 10.3389/fcvm.2022.888593 (PMC9197099; doi:10.3389/fcvm.2022.888593)
Supplement: Supplementary Table 1 — Factors associated with MACE: A multivariate Weibull survival regression. [file Table_1.pdf]

Supplement Table 1. Factors associated with MACE: A multivariate Weibull survival regression

Factors	HR (95% CI)	Robust SE	P-value
Smoking status			
Current smoker	0.92 (0.84, 1.00)	0.042	0.067
Ex-smoker	0.90 (0.83, 0.98)	0.037	0.013
Never	1		
BMI, kg/m^2	0.96 (0.95, 0.97)	0.004	<0.001
CKD			
Yes	1.97 (1.83, 2.11)	0.071	<0.001
No	1		
DM			
Yes	1.21 (1.12, 1.30)	0.044	<0.001
No	1		
Dyslipidemia			
Yes	0.73 (0.68, 0.79)	0.028	<0.001
No	1		
HT			
Yes	1.19 (1.10, 1.29)	0.050	<0.001
No	1		
Cerebrovascular disease			
Yes	1.51 (1.33, 1.70)	0.093	<0.001
No	1		
PAD			
Yes	1.85 (1.53, 2.23)	0.177	<0.001
No	1		
CAD presentation			
STEMI	1.32 (1.15, 1.53)	0.097	<0.001
NSTEMI/unstable Angina	1.41 (1.28, 1.56)	0.072	<0.001
Stable CAD	1		
PCI status			
Emergency	2.60 (2.27, 2.98)	0.179	<0.001
Urgent	1.59 (1.43, 1.78)	0.090	<0.001
Elective	1		
Cardiogenic shock at start of PCI			
Yes	1.93 (1.73, 2.14)	0.104	<0.001
No	1		
IABP			
Yes	1.82 (1.59, 2.08)	0.125	<0.001
No	1		
Extent of CAD			
Left main	1.43 (1.29, 1.59)	0.080	<0.001
TVD	1.08 (0.99, 1.19)	0.051	0.086
DVD	1.08 (0.98, 1.18)	0.051	0.113
SVD	1		

BMI: body mass index, CAD: coronary artery disease, CKD: chronic kidney disease, DM: diabetes mellitus, DVD: double vessel disease, HT: hypertension, IABP: intraaortic balloon pump, MACE: major adverse cardiac event, NSTEMI: non-ST-elevation myocardial infarction, PAD: peripheral arterial disease, PCI: percutaneous coronary

intervention, SD: standard deviation, STEMI: ST-elevation myocardial infarction, SVD: single vessel disease, TVD: triple vessel disease
